# A Case of Jaundice

**Published:** 1883-07

**Authors:** 


### IX.—A CASE OF JAUNDICE.

REPORTED FROM DR. ROBERTSON'S CLINIC.

The patient was a bay horse 16 hands high, aet 12. One year ago this horse had a similar attack. Three days ago showed symptoms of indisposition. Pulse, 56; tongue, furred and of a peculiar greenish yellow, conjunctivæ injected and of a yellow color. General symptoms of lassitude; feces hard and dry; urine scanty with a brick-dust deposit; pain on pressure over loins of right side.

*Treatment.*

Mild aperients, followed by following powders:

℞

Pulv. Feni Exsic., . . . ℥ss.

" Cupri Sulp., . . ℥ii.

" Cinchonæ, . . . ℥i.

" Cantharadis, grs., . xviii.

M̶ et fiat et div in Chart no vi.

Sig. One, twice daily.

To be fed upon mashes and carrots.

Two weeks later the horse was well and at work.

